# Surgical Treatment of Adhesive Capsulitis: A Retrospective Comparative Study of Manipulation Under Anesthesia and/or Capsular Release

**DOI:** 10.7759/cureus.9032

**Published:** 2020-07-06

**Authors:** Bradley Schoch, Daniel Huttman, Usman Ali Syed, Manan S Patel, Mark D Lazarus, Joseph A Abboud

**Affiliations:** 1 Orthopaedics, Mayo Clinic, Jacksonville, USA; 2 Shoulder and Elbow Surgery, Rothman Institute at Thomas Jefferson University, Philadelphia, USA; 3 Shoulder and Elbow Surgery, Rothman Orthopaedic Institute, Philadelphia, USA

**Keywords:** adhesive capsulitis, manipulation under anesthesia, capsular release, frozen shoulder, range of motion, diabetes

## Abstract

Background

No consensus exists among orthopedic surgeons regarding the optimal intervention for adhesive capsulitis. The purpose of this study was to determine which treatment provides the best objective outcome following manipulation under anesthesia (MUA), MUA + arthroscopic capsular release (CR), or CR alone.

Methods

Between 2011 and 2015, 97 shoulders were treated for adhesive capsulitis (MUA, MUA+CR, CR) and followed for three months or until achieving full range of motion (ROM). Patients' charts were reviewed for demographic information, diabetes, pre/post-operative ROM, and complications.

Results

The average age at surgery was 57 years (range: 31-80 years) with a mean follow-up of 6.2 months (range: 2-43 months). ROM improved significantly regardless of treatment modality (p < 0.001). MUA had significantly more external rotation at follow-up than MUA+CR and CR alone (62 vs 49 vs 48, p = 0.02). Groups were similar in regards to post-operative elevation and internal rotation. Loss of external rotation following surgery was significantly more common in the MUA+CR group (p = 0.03). In diabetics, no treatment option was superior to another in regards to final ROM.

Conclusion

Operative treatment of idiopathic adhesive capsulitis is efficacious and safe for improving shoulder ROM across treatment modalities. Surgeon preference may effectively guide treatment independent of diabetic status.

## Introduction

Adhesive capsulitis, also known as frozen shoulder, is stiffening of the shoulder due to scar tissue and is a common cause of shoulder pain affecting 2-5% of the population [1, 2]. The pathologic process of adhesive capsulitis goes through a three-stage progression from initial inflammation and synovitis to fibrosis of the capsule and synovium, ultimately leaving patients with restricted active and passive shoulder range of motion without a clear underlying cause, to resolution in upwards of 24 months [3, 4].

Early on, pain is often a prominent symptom. This can be managed with over the counter analgesics, intraarticular cortisone injections, and rarely narcotics. Stiffness is then managed with a four-quadrant gentle stretching program that can be performed at home or under the supervision of a physical therapist depending on the patient/surgeon preference. This combination has been shown to be effective in 60-95% of patients [5-8].

For a small minority of patients, non-operative management remains ineffective. Risk factors for requiring operative management include diabetes mellitus, more severe initial symptoms, younger at age of onset, and loss of motion despite four months of compliant therapy [9]. If and when the time comes to pursue surgical management, there remains no consensus among the orthopedic community regarding the optimal intervention [10]. Surgical treatment commonly includes manipulation under anesthesia (MUA), an arthroscopic capsular release (CR), or a combination of these two interventions. The purpose of this study was to determine which interventional treatment of adhesive capsulitis provides the best objective outcome. We hypothesized that shoulder range of motion would improve equally among all groups, with a lower complication rate in the arthroscopic only group.

## Materials and methods

After IRB approval at Thomas Jefferson University was obtained, a retrospective chart review was conducted to identify all shoulders undergoing isolated operative treatment (MUA, CR, or MUA with CR) of idiopathic adhesive capsulitis at our institution from the years 2011-2015 utilizing common procedural technology (CPT) codes of 23700 for MUA and 29825 for CR. Shoulders were included if the operative report confirmed primary diagnosis of adhesive capsulitis or frozen shoulder. The decision on which type of operation to perform was left to the sole discretion of the surgeon (ten fellowship-trained Sports and Shoulder & Elbow surgeons). Shoulders with previous surgery or requiring additional procedures at the time of intervention (rotator cuff repair, labral repair, or biceps tenotomy/tenodesis) were eliminated. Ninety-seven shoulders, in 97 patients, met inclusion criteria. Four were lost to follow up and four patients had incomplete preoperative motion measurements. This left 15 shoulders treated with MUA, 38 with CR + MUA, and 44 patients with CR.

Patients' charts were reviewed for age, sex, hand dominance, diabetes, hypothyroidism, pre-operative and post-operative range of motion (forward flexion (FF) in the scapular plane, external rotation with the arm at the side (ER) and internal rotation (IR), and complications). Range of motion was assessed in clinic, subjectively by surgeon. Prior to being offered surgery, all patients had formal physical therapy focusing on passive and active-assisted range of motion in all planes. All patients were followed for a minimum of three months unless full active motion was obtained by the two-month follow-up visit. Full motion was defined as being equal to the contralateral non-affected side, which the patient reported to be normal and free from current pathology.

Surgical technique

All surgical procedures were performed as an outpatient. All patients eligible for nerve blocks were given the option after discussions with the anesthesia staff. Patients were then brought to the operating room and placed under general anesthesia with paralysis. With the patient in the supine position, measurements were taken on both the affected and non-operative sides to confirm restriction of motion that was not secondary to pain while awake. If a manipulation was performed on the affected shoulder it was done utilizing gentle pressure directed by the operative surgeon by grasping the distal humerus and then gently forcing flexion/extension followed by abduction. Force was then applied to the proximal forearm to manipulate the arm in external rotation (at 0 and 90) as well as internal rotation at 90. Final measurements were taken if MUA was an isolated procedure.

For those patients undergoing combined MUA/CR and those undergoing isolated CR, patients were next positioned in the beach chair position. MUA was performed prior to CR. Following standard prep and draping, an arthroscope was inserted into the shoulder and a diagnostic arthroscopy was performed to confirm no other source of pathology within the shoulder joint. A circumferential release was performed of the superior capsule, rotator interval, anterior capsule, posterior, and finally the inferior capsule. Releases were performed with a combination of radiofrequency probes, shavers and biters, per surgeon preference. Upon completion of the intraarticular releases, the scope was moved to the subacromial space. A subacromial bursectomy was performed in order to visualize the rotator cuff and again confirm no associated pathology and make sure any subacromial adhesions were released.

All patients were discharged from the hospital with a soft sling in place. Formal physical therapy (PT) was initiated in the first 24-48 hours, two-three times per week with home-based exercises between visits. PT progressed from passive and active stretching to passive and active strengthening. Patients returned to clinic at two weeks for a wound check and to assess range of motion. Those noted to be progressing slowly were brought back in two weeks, whereas those maintaining their motion were allowed to follow up five weeks postoperatively. Patients were followed for a minimum of three months or until full motion was obtained in the absence of pain.

Statistical analysis

The overall data for all patients undergoing surgical treatment was analyzed for differences in pre-operative and post-operative range of motion using a student t-test. These patients were then divided into three groups (MUA, MUA + CR, CR). The subgroups were analyzed using an ANOVA test for continuous variables, a Kruskal-Wallis test for ordinal variables, and a chi-squared test for nominal variables. A post hoc Tukey test performed for ANOVA tests was found to be significant. The alpha level for all tests was set at 0.05.

## Results

A total of 97 shoulders, in 28 males and 69 females, were examined following surgical treatment for adhesive capsulitis during the study period. The dominant arm was affected in 41% of shoulders. The average age at the time of surgery was 57 years (range: 31-80 years). Follow-up averaged 6.2 months (range: 2-43 months). Overall, patients achieved significant improvements in all ranges of motion. Forward flexion improved from an average of 106 to 156 (p < 0.001). Mean external rotation improved from 26 to 50 (p < 0.001). Mean internal rotation improved from the sacrum to mid lumbar spine (p < 0.001).

When examining individual treatment groups, all groups were similar in regards to age and preoperative diagnoses of diabetes and hypothyroidism (Table 1). The MUA+CR group had greater pre-op IR; however, all were still to the lumbar spine despite the significant p-value (p = 0.006). Groups had similar pre-op in regards to forward elevation and external rotation. Post-operatively, the MUA group had the greatest external rotation (p = 0.02). IR was similar amongst all groups following surgery. However, when looking at only change in motion, all groups were similar in all measured motions (p > 0.05). Full results can be seen in Table 1.

**Table 1:** Assessment of pre- and post-operative range of motion.

	CR (range) n = 44	MUA + CR (range) n = 38	MUA (range) n = 15	p-value	CR vs CR+MUA	CR vs MUA	MUA vs CR+MUA
Age	58 (31-79)	55 (42-65)	59 (36-80)	0.06			
Sex (M/F)	3/12	13/25	12/32	0.6			
Diabetes	13	5	3	0.2			
Hypothyroidism	7	7	3	0.9			
Pre-Op							
Forward Elevation	109 (20-160)	107 (30-180)	94 (20-150)	0.3			
External Rotation	25 (-10-60)	27 (-10-70)	31 (0-70)	0.5			
Internal Rotation	1.23 (1-3)	1.49 (1-3)	1.21 (0-3)	0.006	0.03	0.6	0.02
Post-Op							
Follow up	5.8 (2-35)	5.0 (2-21)	9.9 (2-43)	0.07			
Forward Elevation	158 (110-180)	156 (110-175)	153 (80-175)	0.7			
External Rotation	48 (10-80)	49 (25-70)	62 (30-80)	0.02	0.9	0.02	0.05
Internal Rotation	2.22 (1-3)	2.49 (1-3)	2.10 (1-3)	0.08			
Change in motion							
Forward Elevation	49 (0 to +130)	48 (-45 to +100)	59 (-45 to 150)	0.6			
External Rotation	24 (-15 to +55)	23 (-15 to +50)	30 (-10 to +80)	0.5			
Internal Rotation	1.02 (0 to 2)	1.065 (1 to 2)	1.062 (-2 to 2)	0.2			

Compared to before surgery, 85% of patients demonstrated improvements in all planes of motion. Fifteen percent of patients demonstrated loss of motion in at least one plane of motion. External rotation was most commonly lost (nine shoulders) followed by internal rotation (four shoulders) and forward elevation (three shoulders). There was no difference among treatment groups in regards to loss of forward elevation or internal rotation (p = 0.2 and p = 0.8, respectively). Loss of external rotation was most common in the MUA+CR group (18%) vs MUA (7%) and CR (2%) (p = 0.03).

When attempting to compare those patients with diabetes (n = 21) to those without, we were unable to identify any significant differences in pre, post, or change in range of motion (p > 0.5). No specific treatment (MUA, MUA+CR, CR) provided additional benefit in this group of patients.

## Discussion

Operative treatment of idiopathic adhesive capsulitis is efficacious and safe for improving shoulder range of motion (ROM) regardless of the surgical treatment method chosen. All of the studied treatment methods demonstrated the ability to restore range of motion to the affected shoulder after failure of non-operative management. Surgeons continue to debate the merits of MUA compared to CR, with some attempting to combine both an MUA and CR [11]. Few studies have directly compared these treatment techniques head to head [12-14]. There remains no consensus amongst the orthopedic community as to which surgical treatment is preferred. Our results support that one technique is not superior to another in improving range of motion.

Similar to other studies we found that range of motion improved significantly, no matter the treatment type [13]. There was no statistically significant difference between groups. Pre-operative internal was noted to be significantly lower in the MUA and CR groups; however, no difference in post-operative range of motion or change in motion was noted between groups at follow-up. Post-operative external rotation was significantly better in the MUA group. However, the clinical significance of these findings remains debatable, especially with IR where all groups were able to reach the mid lumbar spine.

Diabetics have also received additional attention due to their documented risk of recurrence and concern that MUA is not adequate [15, 16]. Massoud et al. reported on 47 shoulders in patients with diabetes treated with MUA, or MUA+CR. CR was performed only when range of motion could not be restored with isolated manipulation. Their results showed significant improvements in AROM, similar to our results. Massoud et al. recommended MUA be performed in diabetics; however, no direct comparison was made between ROM outcomes in those treated with MUA alone or those who did not improve with MUA and required subsequent arthroscopic CR. When comparing patients with diabetes to those without, we were unable to identify any difference in final range of motion for the group overall or between groups (MUA, MUA+CR, CR). This suggests that MUA is not superior to CR on the basis of range of motion. Ogilvie-Harris et al. similarly compared MUA to CR and found no difference in diabetic and non-diabetic in regards to range of motion following surgical intervention [13].

Concern also remains regarding MUA and the potential for soft tissue damage to the shoulder. Previous reports have demonstrated complications following MUA including: labral tearing, rotator cuff tears, dislocation, nerve injury, and fracture [17-21]. In our series, no patient undergoing MUA or MUA+CR sustained detectable iatrogenic injuries to the shoulder soft tissue structures. This is significantly lower than the 30% injury rate reported by Loew et al. [19]. This may be technique dependent, patient selection, or simply due to the patient sample size. Additionally, it remains possible some patients undergoing MUA had injuries that did not manifest clinically. No visible damage was seen in 55 shoulders undergoing MUA followed by CR.

The strength of this study is that it represents the first study directly comparing the three surgical treatment modalities used most commonly by orthopedic surgeons to treat adhesive capsulitis. It also represents one of the largest series of patients undergoing these procedures. The study remains limited by its retrospective nature. Due to the inclusion of multiple surgeons, exact operative indications and operative techniques varied. Length of preoperative symptoms was also not assessed due to our large referral base from our non-operative practice. While all patients failed formal physical therapy prior to surgery, no standardized therapy program or treatment duration was utilized. Additionally, no non-operative control group was assessed. Patients undergoing MUA alone were underrepresented compared to those undergoing MUA/CR or CR. The MUA/CR group was also subject to selection bias, given that some surgeons proceeded to CR only after MUA had failed to provide adequate increases in ROM. This more resistant group still had outcomes similar to the other treatment modalities at follow-up. Another weakness is that this study did not obtain pain scores, patient reported outcomes or long-term clinical outcomes. Despite this, historical references have shown reliable pain relief and maintenance of range of motion gains/patient satisfaction over time [22, 23].

## Conclusions

This study represents the largest series of patients undergoing surgical treatment of adhesive capsulitis with a direct comparison between MUA, MUA/CR, and CR alone. All interventions significantly improved ROM with no identifiable complications, however, the MUA group had the greatest external rotation, postoperatively. Based on this data, adhesive capsulitis can be treated successfully with any of the three modalities, regardless of diabetic or hyperthyroid status, with stronger improvements in external rotation with MUA only. There remains a need for further studies comparing these treatments in a prospective manner to confirm these findings, as well as explore the functional impact of surgical intervention.
